# Comparison of the Outcomes of Aortic Valve Replacements (AVRs) Performed via Conventional Full Sternotomy and Upper Mini Sternotomy: Our Experience at Hayatabad Medical Complex, Pakistan

**DOI:** 10.7759/cureus.73278

**Published:** 2024-11-08

**Authors:** Muhammad Aasim, Raheela Aziz, Atta ul Mohsin, Raheel Khan, Gulshad Aziz, Jibran Ikram

**Affiliations:** 1 Department of Cardiac Surgery, Hayatabad Medical Complex, Peshawar, PAK; 2 Department of Cardiac Surgery, Khyber Girls Medical College (KGMC), Peshawar, PAK; 3 Department of Anatomy, Khyber Medical University (KMU) Institute of Dental Sciences, Kohat, PAK; 4 Department of Cardiovascular Medicine, Hayatabad Medical Complex, Peshawar, PAK

**Keywords:** aortic valve replacement, cardiopulmonary bypass, conventional sternotomy, mini-avr, upper mini-sternotomy

## Abstract

Introduction

Aortic valve replacement (AVR) for severe symptomatic aortic stenosis is a commonly performed procedure, yielding excellent long-term outcomes. Comparing a mini sternotomy with a conventional sternotomy is essential to evaluate less invasive options that can improve patient recovery and reduce postoperative complications. This insight supports surgical decision-making for better AVR patient outcomes.

Methodology

This retrospective comparative study aims to compare clinical outcomes between mini sternotomy for aortic valve replacement (mini-AVR) and conventional full sternotomy for aortic valve replacement (FS-AVR). Patient records of isolated AVR from January 2021 to July 2023 were reviewed, excluding those with comorbidities or requiring concomitant procedures. Outcomes measured included sternal wound infections, operative time, length of ICU stay, cardiopulmonary bypass (CPB) time, and aortic cross-clamp time.

Results

The study included 65 patients (47 males and 18 females). Among the participants, 30 patients underwent AVR using full sternotomy, while 35 patients had the procedure performed via upper mini-sternotomy. The mini-AVR group experienced significantly less bleeding and a reduced need for blood transfusions compared to the FS-AVR group. Additionally, patients in the FS-AVR group had longer ICU stays and prolonged ventilation times. Notably, in contrast to findings from other studies, our research revealed that CPB time and aortic cross-clamp time were shorter in the mini-AVR group.

Conclusion

Mini-sternotomy has proven to be a safe and effective approach for AVR, with the mini-AVR group experiencing fewer complications, such as reduced bleeding and decreased need for blood transfusions. Additionally, patients benefit from shorter ICU stays, reduced ventilation time, and quicker overall recovery.

## Introduction

Aortic valve degeneration affects 2%-7% of people aged 65 or older and ranks as the fourth most prevalent cardiovascular condition globally [1]. Its pathology includes processes similar to those in atherosclerosis, including lipid accumulation, inflammation, and calcification [2]. Aortic valve replacement (AVR) is the procedure of choice for severe aortic valve disease and generally improves the long-term survival of patients [3, 4]. In 1996, minimally invasive surgical (MIS) AVR was reported to offer several benefits over conventional full sternotomy (FS) procedures, such as better cosmetic outcomes, reduced pain, reduced surgical trauma, decreased blood loss, earlier functional recovery, and a shorter hospital stay [5]. Since the mid-1990s, several minimally invasive approaches for aortic valve operations have been introduced and used with increasing frequency: upper partial sternotomy [6,7], anterolateral right mini-thoracotomy [8], right parasternal approach [9], and transverse sternotomy [10]. Nowadays, the right anterior thoracotomy and upper partial sternotomy are the predominant approaches for mini sternotomy for aortic valve replacement (mini-AVR) [11]. Mini-AVR has been criticized for increased cardiopulmonary bypass, cross-clamp time, operating room time, and higher cost [12, 13]. Because partial sternotomy or thoracotomy provides limited exposure to the heart, myocardial protection, de-airing, and valve exposure can be more challenging [14].

## Materials and methods

In this retrospective comparative study, we collected data manually from the patients’ files who were operated on for AVR surgery in the cardiac surgery department of Hayatabad Medical Complex, Peshawar, Pakistan. All the operated patients’ files were reviewed for the determined study period. A total of 65 patients were identified with similar characteristics who were operated on for isolated AVR during this study period, from January 2021 to July 2023. Patients with debilitating comorbidities and patients undergoing concomitant surgeries for other valvular, myocardial, or coronary lesions were excluded to curtail the confounding factors in the study. Two groups were made; in one group we put patients undergoing AVR with conventional full sternotomy (FS-AVR), and in the other group patients undergoing AVR with upper mini sternotomy (mini-AVR) were placed. Variables were recorded, and data were analyzed by Pearson's chi-square test appropriately using IBM SPSS Statistics software, version 20 (IBM Corp., Armonk, NY).

Surgical techniques

Mini Sternotomy for Aortic Valve Replacement

**Figure 1:**
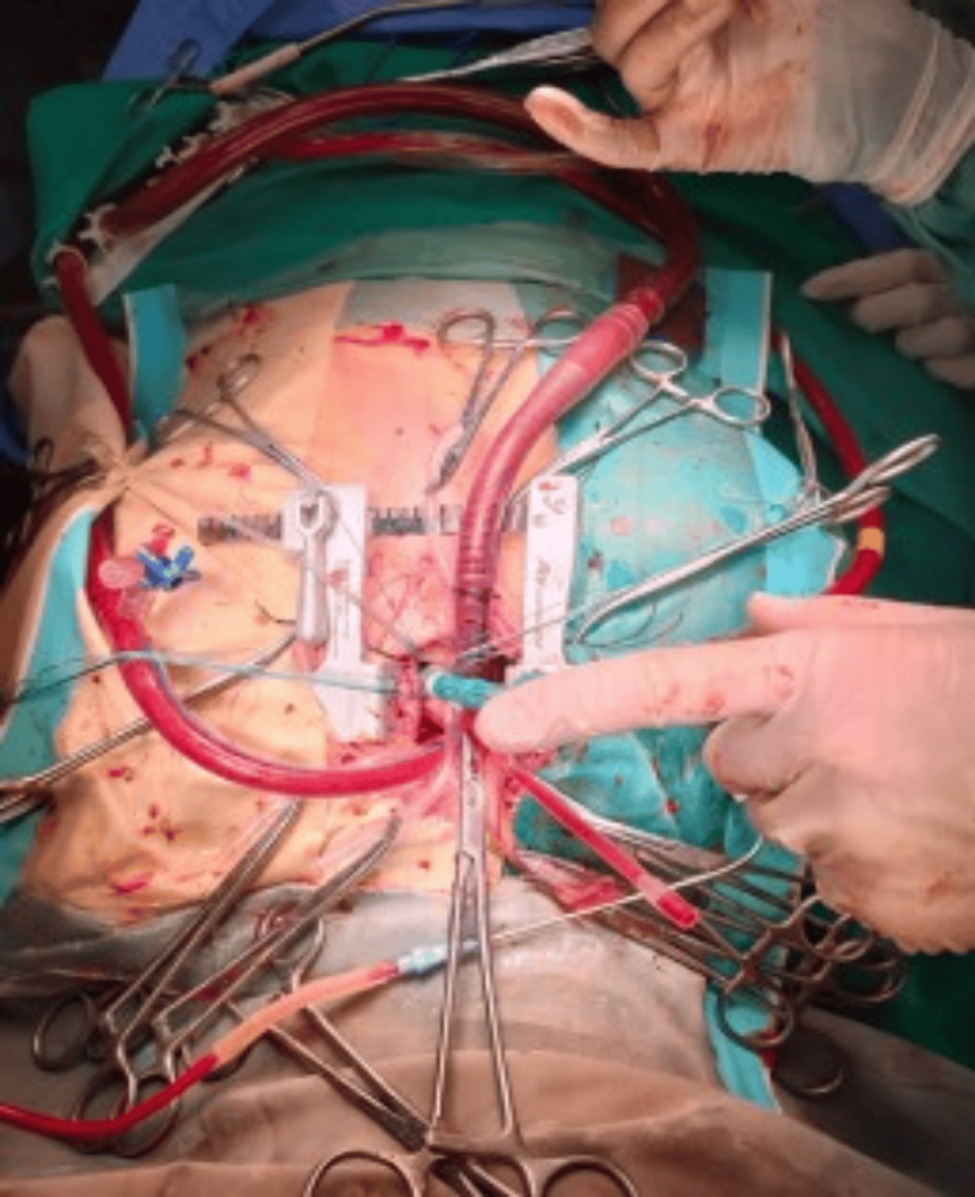
Intraoperative view of mini-AVR, showing CPB cannula arrangements and insertion of the prosthetic aortic valve using interrupted sutures. mini-AVR: mini sternotomy for aortic valve replacement; CPB: cardiopulmonary bypass

A minimally invasive approach was used with a small midline skin incision from the suprasternal notch to the 3^rd^/4^th^ intercostal space for mini sternotomy. The sternum was exposed and cut vertically through the manubrium and carefully transected horizontally at the 3^rd^/4^th^ intercostal space in a standard J-shaped partial sternotomy, avoiding damage to the right internal mammary artery. An appropriate-size single-blade sternal spreading retractor was used before thymic fat was dissected, followed by pericardiotomy and placement of pericardial sutures. The patient was fully heparinized, and aortic and right atrial cannulation (conventional cardiopulmonary bypass circuit) was performed to establish cardiopulmonary bypass (CPB). Cardiac arrest was induced using antegrade cardioplegia. Aortotomy was performed to excise the diseased aortic valve, and replacement was done with the latest generations of double leaflet mechanical aortic valve prosthesis, followed by closure of the aortotomy. Suction de-airing was carried out through a standard aortic root cannula placed at an appropriate site on the ascending aorta, distal to the aortotomy incision, and proximal to the aortic cross-clamp site (Figure 1).

**Figure 2:**
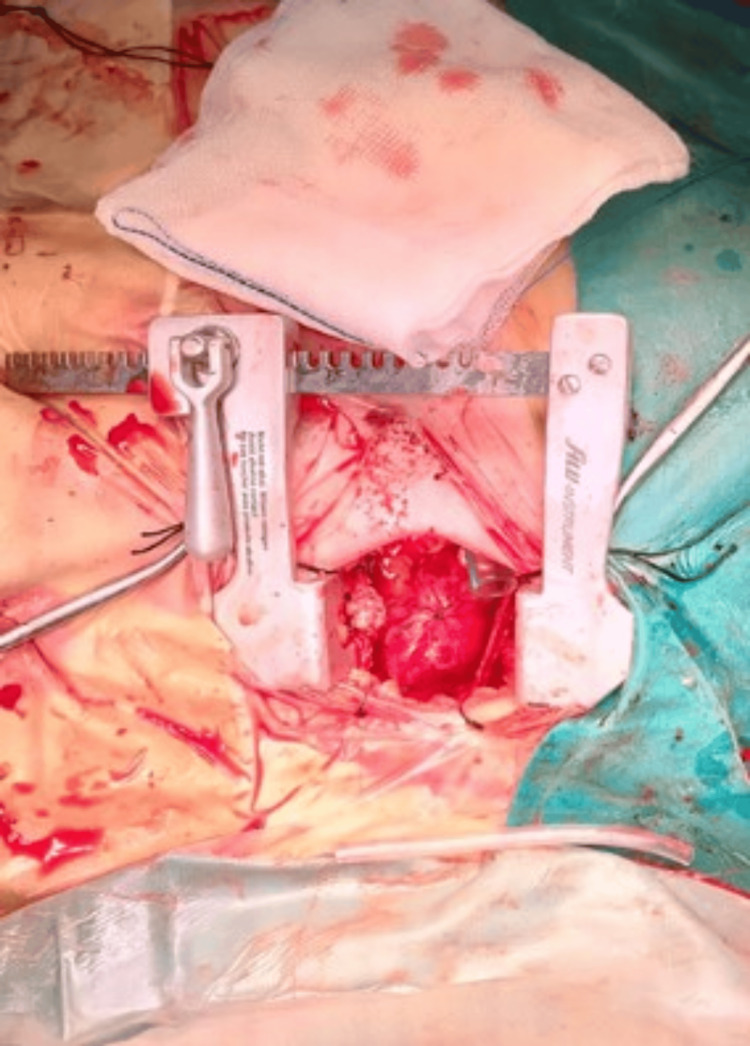
Intraoperative view showing completed mini-AVR procedure, aorta closed, decannulated off-CPB, and pericardial drain placed in. mini-AVR: mini sternotomy for aortic valve replacement; CPB: cardiopulmonary bypass

We used all the conventional instruments and CPB cannulas and circuits. Epicardial pacing wire was placed before taking the cross-clamp off. Routine rewarming was carried out on CPB, mediastinal drains were placed as indicated, and off-CPB decannulation was performed in the standard way. Less number of steel wires are required for closing the upper partial sternotomy (Figure 2).

Full Sternotomy for Aortic Valve Replacement

Full sternotomy AVR is a well-established, long-practiced surgical technique for AVR. The cutting of the whole length of the sternum and a bigger incision is the main difference between this conventional AVR procedure as compared to the upper partial sternotomy mini-AVR.

## Results

The study included 65 patients, 30 patients in the FS-AVR group, and 35 patients in the mini-AVR group. Out of the total patients, 47 were males (20 in FS-AVR and 27 in mini-AVR) and 18 were females (10 in FS-AVR and eight in mini-AVR), with the mean age in mini-AVR group patients being 41.09±15.4years and in the FS-AVR group patients 47.5±17.4years. The patients were normally distributed in the groups by age and gender, and no statistical difference was observed between the groups. All these procedures were performed using CPB machine support. All patients underwent AVR with a mechanical prosthesis. Analysis of the intraoperative data revealed a statistically insignificant (p-value 0.1 and 0.4, respectively) longer duration of cardiopulmonary bypass and cross-clamp time in the conventional FS-AVR group as compared to the mini-AVR group. Perioperative parameters, like blood transfusions, drainage, and ventilation in hours, were less in the mini-AVR group as compared to the FS-AVR group. The difference in ventilation times in hours and requirements for blood transfusion were less in favor of the mini-AVR group, reaching a statistically significant level with a p-value of 0.02 and 0.001, respectively. Preoperative parameters are compared in Table 1.

**Table 1:** Preoperative parameters; comparison of conventional FS-AVR group and mini-AVR group (n=65) FS-AVR: full sternotomy for aortic valve replacement; mini-AVR: mini sternotomy for aortic valve replacement

Parameters	FS-AVR, n (%)	Mini-AVR, n (%)	Total N (%)	p-value
Male	20 (30.8)	27 (41.5)	47 (72.3)	0.3
Female	10 (15.4)	8 (12.3)	18 (27.7)	
Mean Age ± S.D	47.5±17.4	41.09±15.4		0.1
Hypertension positve (+ve)	18 (27.7)	13 (20.0)	31 (47.7)	0.06
Smoker +ve	1 (1.5)	3 (4.6)	4 (6.2)	0.3
Diabetes mellitus+ve	9 (13.8)	5 (7.7)	14 (21.5)	0.2

Perioperative parameters are detailed in Table 2 for further comparison.

**Table 2:** Perioperative parameters; comparing the FS-AVR group with the mini-AVR group (n=65) FS-AVR: full sternotomy for aortic valve replacement; mini-AVR: mini sternotomy for aortic valve replacement

Parameters	FS-AVR, n (%)	Mini-AVR, n (%)	Total N (%)	p-value
Re-intubation	1 (1.5)	0 (0.0)	1 (1.5)	0.2
Ventilator time in hours	9.1±7.0	5.7±2.8		0.02
ICU stay days	3±1.0	2±0		0.06
Drain	915.6±733	655±457		0.08
Re-exploration	1 (1.5)	0 (0.0)	1 (1.5)	0.2
Blood transfusion: 500ML	4 (6.2)	4 (6.2)	8 (12.3)	0.001
Blood transfusion: 501ML-1000Ml	9 (13.8)	0 (0.0)	9 (13.8)	
Blood transfusion: 1001Ml-2000Ml	1 (1.5)	0 (0.0)	1 (1.5)	
Blood transfusion: >2000Ml	2 (3.1)	0 (0.0)	2 (3.1)	
Wound infection	1 (1.5)	0 (0.0)	1 (1.5)	0.2
In-hospital mortality	1 (1.5)	0 (0.0)	1 (1.5)	0.2

Sternal wound infection was observed in one patient of the FS-AVR group. There was one in-hospital mortality in the FS-AVR group, while no mortality was observed in the mini-AVR group. There were no instances of conversion from mini-sternotomy to full sternotomy in the mini-AVR group. Patients who underwent AVR through median sternotomy had longer hospital and ICU stays compared to those in the mini-AVR group.

## Discussion

The conventional approach for aortic valve surgery has long relied on median sternotomy, a technique that provides extensive access to the thoracic cavity and allows for clear visualization and handling of the aortic valve. This standard approach, however, is associated with considerable trauma to the chest wall, extended postoperative pain, and a lengthy recovery period. In recent years, advances in surgical techniques and instrumentation have led to the adoption of minimally invasive procedures, most notably mini sternotomy, as an alternative to traditional full sternotomy. Unlike the conventional method, mini sternotomy requires a smaller incision and results in less disruption to the surrounding musculoskeletal and vascular structures. This reduction in surgical trauma not only minimizes the physical burden on patients but also preserves the structural stability of the sternum and thorax, which is crucial for a swift postoperative recovery.

Mini sternotomy has several notable advantages beyond reduced trauma. The smaller incision and minimized tissue dissection are associated with faster wound healing and shorter hospital stays, which can improve overall patient satisfaction and reduce healthcare costs. Additionally, the limited disruption to the chest wall and muscular structures results in improved cosmetic outcomes, a benefit that has become increasingly important to patients seeking less invasive surgical options. Mini sternotomy has also been shown to lower the risk of postoperative complications, particularly sternal wound infections, which are a serious concern in full sternotomy due to the larger surgical exposure and increased risk of bacterial contamination. By preserving the structural integrity of the sternum, mini sternotomy not only supports a quicker recovery but also substantially decreases the likelihood of sternal instability and wound-related complications. [15]. Mini sternotomy is linked to reduced postoperative bleeding, resulting in less tissue trauma and a lower inflammatory response [1]. In a meta-analysis by Khoshbin et al., no significant difference in blood loss was found between mini sternotomy and full sternotomy in randomized controlled trials [16]. However, Filip et al. reported a 230 ml drainage difference between the two groups [17]. Our study revealed a significant difference of 260 ml between the two groups, emphasizing the importance of this finding, which leads to fewer blood transfusions in the mini-AVR group.

The duration of hospital stay is a crucial factor in predicting morbidity and mortality. Our study observed that patients undergoing FS-AVR had a longer ICU stay compared to those in the mini-AVR group. Khoshbin et al. (2011) reported a statistically significant (p = 0.003) prolongation of ICU stay for patients who underwent full sternotomy (16), confirming the importance of this finding. There was only one mortality, and that was in the FS-AVR group. Raja et al. also presented a lower mortality rate in the mini-AVR group than in the full sternotomy group, but without statistical significance [18]. Nonetheless, mini sternotomy is technically demanding and requires refined surgical skills to overcome the limited visual and physical access to the surgical field. This constraint could lead to variable outcomes depending on the surgeon's experience. Our findings suggest that while mini sternotomy is safe and effective in experienced hands, broader implementation in centers with less surgical expertise may require additional training to minimize potential complications and optimize patient outcomes.

Our study has limitations that must be acknowledged. Firstly, this was a retrospective analysis conducted at a single center with a relatively small sample size, which may affect the generalizability of the findings. Additionally, although we matched patients based on preoperative characteristics, potential selection bias cannot be entirely ruled out. Finally, the study lacks long-term follow-up data, preventing an assessment of the durability and long-term outcomes of mini-AVR compared to full sternotomy. Future multicenter studies with larger patient cohorts and extended follow-up are necessary to further validate our findings and better understand the long-term benefits and risks of mini-AVR.

## Conclusions

Our study concludes that mini sternotomy for AVR is both safe and effective, with fewer complications observed in the mini-AVR group compared to the conventional FS-AVR group. Additionally, patients in the mini-AVR group benefited from improved cosmetic outcomes. Consistent with existing literature, our findings suggest that mini-sternotomy reduces ICU stay, presenting a cost-effective alternative. However, the technique demands a higher level of surgical expertise compared to a full sternotomy. Interestingly, unlike most studies, we found that operative time was shorter with mini-AVR. In conclusion, mini-sternotomy is a reliable procedure that does not prolong aortic cross-clamp or cardiopulmonary bypass times, offering a viable and secure option for AVR.

## References

[1] Vahanian A, Alfieri O, Andreotti F (2012). Guidelines on the management of valvular heart disease (version 2012). Eur Heart J.

[2] Otto CM, Kuusisto J, Reichenbach DD, Gown AM, O'Brien KD (1994). Characterization of the early lesion of 'degenerative' valvular aortic stenosis. Histological and immunohistochemical studies. Circulation.

[3] Lund O, Nielsen SL, Arildsen H, Ilkjaer LB, Pilegaard HK (2000). Standard aortic St. Jude valve at 18 years: performance profile and determinants of outcome. Ann Thorac Surg.

[4] Lund O, Chandrasekaran V, Grocott-Mason R (1999). Primary aortic valve replacement with allografts over twenty-five years: valve-related and procedure-related determinants of outcome. J Thorac Cardiovasc Surg.

[5] Murtuza B, Pepper JR, Stanbridge RD, Jones C, Rao C, Darzi A, Athanasiou T (2008). Minimal access aortic valve replacement: is it worth it?. Ann Thorac Surg.

[6] Cosgrove DM 3rd, Sabik JF (1996). Minimally invasive approach for aortic valve operations. Ann Thorac Surg.

[7] Svensson LG (1997). Minimal-access "J" or "j" sternotomy for valvular, aortic, and coronary operations or reoperations. Ann Thorac Surg.

[8] Benetti F, Rizzardi JL, Concetti C, Bergese M, Zappetti A (1999). Minimally aortic valve surgery avoiding sternotomy. Eur J Cardiothorac Surg.

[9] Cohn LH (1998). Minimally invasive aortic valve surgery: technical considerations and results with the parasternal approach. J Card Surg.

[10] Bridgewater B, Steyn RS, Ray S, Hooper T (1998). Minimally invasive aortic valve replacement through a transverse sternotomy: a word of caution. Heart.

[11] Malaisrie SC, Barnhart GR, Farivar RS (2014). Current era minimally invasive aortic valve replacement: techniques and practice. J Thorac Cardiovasc Surg.

[12] Cooley DA (2000). Antagonist's view of minimally invasive heart valve surgery. J Card Surg.

[13] Cunningham MJ, Berberian CE, Starnes VA (2011). Is transthoracic minimally invasive aortic valve replacement too time-consuming for the busy cardiac surgeon?. Innovations (Phila).

[14] Tabata M, Umakanthan R, Khalpey Z, Aranki SF, Couper GS, Cohn LH, Shekar PS (2007). Conversion to full sternotomy during minimal-access cardiac surgery: reasons and results during a 9.5-year experience. J Thorac Cardiovasc Surg.

[15] Litwinowicz R, Bryndza M, Chrapusta A, Kobielska E, Kapelak B, Grudzień G (2016). Hyperbaric oxygen therapy as additional treatment in deep sternal wound infections - a single center's experience. Kardiochir Torakochirurgia Pol.

[16] Khoshbin E, Prayaga S, Kinsella J, Sutherland FW (2011). Mini-sternotomy for aortic valve replacement reduces the length of stay in the cardiac intensive care unit: meta-analysis of randomised controlled trials. BMJ Open.

[17] Filip G, Bryndza MA, Konstanty-Kalandyk J (2018). Ministernotomy or sternotomy in isolated aortic valve replacement? Early results. Kardiochir Torakochirurgia Pol.

[18] Raja SG, Benedetto U, Amrani M (2013). Aortic valve replacement through J-shaped partial upper sternotomy. J Thorac Dis.

